# No Place Like Home: Cross-National Data Analysis of the Efficacy of Social Distancing During the COVID-19 Pandemic

**DOI:** 10.2196/19862

**Published:** 2020-05-28

**Authors:** Dursun Delen, Enes Eryarsoy, Behrooz Davazdahemami

**Affiliations:** 1 Center for Health Systems Innovation Department of Management Science and Information Systems Oklahoma State University Tulsa, OK United States; 2 School of Management Sabanci University Istanbul Turkey; 3 Department of IT and Supply Chain Management University of Wisconsin-Whitewater Whitewater, WI United States

**Keywords:** COVID-19, public health, social distancing, machine learning, pandemic

## Abstract

**Background:**

In the absence of a cure in the time of a pandemic, social distancing measures seem to be the most effective intervention to slow the spread of disease. Various simulation-based studies have been conducted to investigate the effectiveness of these measures. While those studies unanimously confirm the mitigating effect of social distancing on disease spread, the reported effectiveness varies from 10% to more than 90% reduction in the number of infections. This level of uncertainty is mostly due to the complex dynamics of epidemics and their time-variant parameters. However, real transactional data can reduce uncertainty and provide a less noisy picture of the effectiveness of social distancing.

**Objective:**

The aim of this paper was to integrate multiple transactional data sets (GPS mobility data from Google and Apple as well as disease statistics from the European Centre for Disease Prevention and Control) to study the role of social distancing policies in 26 countries and analyze the transmission rate of the coronavirus disease (COVID-19) pandemic over the course of 5 weeks.

**Methods:**

Relying on the susceptible-infected-recovered (SIR) model and official COVID-19 reports, we first calculated the weekly transmission rate (*β*) of COVID-19 in 26 countries for 5 consecutive weeks. Then, we integrated these data with the Google and Apple mobility data sets for the same time frame and used a machine learning approach to investigate the relationship between the mobility factors and *β* values.

**Results:**

Gradient boosted trees regression analysis showed that changes in mobility patterns resulting from social distancing policies explain approximately 47% of the variation in the disease transmission rates.

**Conclusions:**

Consistent with simulation-based studies, real cross-national transactional data confirms the effectiveness of social distancing interventions in slowing the spread of COVID-19. In addition to providing less noisy and more generalizable support for the idea of social distancing, we provide specific insights for public health policy makers regarding locations that should be given higher priority for enforcing social distancing measures.

## Introduction

As of mid-May 2020, approximately 4.5 million people worldwide have been infected by the new deadly coronavirus disease (COVID-19) [1]. In the absence of a vaccine or effective medication, public health experts and epidemiologists suggest that social distancing is the most effective intervention to control the spread of the disease or “flatten the curve” [2,3]. Based on this concept, some serious restrictive policies (eg, shutting down businesses and closing schools) have been enacted by the governments of the affected countries to encourage (and, in some countries, to force) people to stay at home*.*

The effectiveness of social distancing in response to an epidemic has been widely studied, mostly using simulation-based methods. For example, using a differential game approach, Reluga [4] argues that optimal social distancing can only reduce the chance of infection by less than 30%. In another agent-based simulation study using a small population, Kelso et al [5] showed that depending on the initial reproduction number (*R*_0_) of the epidemic and the delay from the first case until the introduction of social distancing measures, the attack rate of the disease can be reduced by between 10% and 73%. Ahmed et al [6], in a systematic review of prior research, stated that social distancing measures in workplaces caused a median reduction of 23% in the cumulative H1N1 influenza attack rate during the 2009 pandemic. In another study, Earn et al [7] showed that school closure had a considerable mitigating effect on the incidence of pandemic influenza in Alberta, Canada. Also, multiple studies have discussed the effects of social distancing on the 1918 influenza pandemic [8-10].

With respect to the COVID-19 pandemic, some recent studies have discussed the effects, challenges, and consequences of social distancing policies. Andersen [11], for instance, shows that mandatory social distancing measures have been effective in reducing visits to public locations. Additionally, Kissler et al [12] maintain that while social distancing is effective, intermittent social distancing should be continued until 2022 to fully control the epidemic. Similarly, Singh and Adhikari [13] propose that a 3-week lockdown is insufficient for controlling the disease in India and that intermittent social distancing should remain in place. In a simulation-based study, Koo et al [14] showed that under scenarios of different *R*_0_ values of COVID-19 (1.5, 2, or 2.5) and social distancing interventions (combinations of quarantine, school closure, and distance working), the number of infections may be reduced by 78.2%-99.3%. Another simulation study in Australia shows that infected case isolation is the most effective social distancing intervention among others (ie, school closure, distance working, and community contact reduction) [15]. Using an online questionnaire approach, Luo et al [16] showed that social distancing policies were effective in containing the spread of COVID-19 from Wuhan City to other areas of China. Greenstone and Nigam [17] estimated that social distancing measures in the United States would save 1.7 million lives by October 2020, and the monetary mortality benefit involved is around US $8 trillion.

Recently, particularly since the spread of COVID-19, researchers have begun to utilize geolocation data obtained from navigation and tracking information systems to analyze the consequences of social distancing policies. For example, using GPS data, Engle et al [18] showed that a higher perceived prevalence of COVID-19 in a small US community (from 0% to 0.003%) reduced mobility by 2.31%. Additionally, Queiroz et al [19] used cell phone navigation data of millions of people in Sao Paulo to show that mandatory social distancing measures have effectively changed the mobility patterns of people in the largest city in Brazil. A similar study was performed by Warren and Skillman [20] to study mobility changes in the United States in response to COVID-19. In another study, Gibson and Rush [21] used data from a geographic information system to discuss the feasibility of implementing social distancing in informal settlements in Cape Town.

Simulation-based studies have consistently shown the overall mitigating role of various social distancing interventions in the spread of epidemics. However, due to the complexity and time-variant nature of diseases, the reported effectiveness of interventions in these studies varies greatly and, in most cases, relies on local assumptions; hence, the results are not generalizable.

Recently, Google LCC [22] and Apple Inc [23] published data sets indicating changes in mobility (compared to an average baseline before the COVID-19 pandemic) of people in different categories of places (eg, transit stations and grocery stores) and different types of activities (eg, driving and walking) based on GPS data collected from users of their navigation applications around the world. These reports confirm the effectiveness of government incentives and restrictive policies to make people stay at home by indicating considerable decreases in mobility within public places (and, in turn, increases in mobility within residential areas); however, the effectiveness of these measures in slowing the disease spread is not apparent. Particularly, many countries are still experiencing increasing numbers of confirmed COVID-19 cases despite having social distancing policies in effect for several weeks; this raises the question of to what extent, if any, the changes in mobility patterns resulting from these policies were effective in managing the disease spread. In this study, we seek to clarify this issue.

To this end, we relied on the susceptible-infected-recovered (SIR) model, one of the most common compartmental models in studying epidemics, along with official reports on the number of COVID-19 cases in different countries to estimate the average transmission rate (*β*) of the disease. While the original SIR model considers a time-invariant *β* value, intuitively, the speed of the epidemic can be at least partially manipulated over time; thus, the magnitude of the parameter *β* can be time-variant (Katriel and Stone [24]; Liu et al [25]). Therefore, each estimation pertaining to a different time section (weeks, in our study) may yield a different *β* value. In our study, these varying *β* values correspond to the weekly mobility statistics with a 7-day lag (considered to reflect the effect of mobility changes on the disease transmission rate). The resulting data set was used to train a machine learning regression algorithm to investigate the relationship between mobility and disease transmission. To the best of our knowledge, this is the first study that uses real transactional data to investigate the actual contribution of social distancing policies (through mobility reduction) in controlling the spread of a pandemic.

## Methods

### Data Sources

#### Google and Apple Mobility Data Sets

In April 2020, Google LLC [22] and Apple Inc [23] started sharing daily mobility data from select regions and select countries in the world. The Google data set incorporates five different mobility trend variables: grocery and pharmacy (supermarkets, farmer’s markets, drug stores, and pharmacies), parks (national/local parks, public beaches, and gardens), transit stations (public transport hubs, including train, bus, and subway stations), retail and recreation (restaurants, cafés, shopping centers, movie theaters), residential (places of residence), and workplaces. The data sets show trends from prior to the outbreak (Google does not provide any specific benchmark date) onward. The Apple data set also shows the relative volume of requests for directions compared to a specific baseline volume of January 13, 2020. Google and Apple do not include mobility data on some countries in the top 30 in terms of cumulative cases of COVID-19, such as Russia, China, the United Kingdom, Iran, and Algeria. Therefore, our analysis is limited to the countries included in both the European Centre for Disease Prevention and Control (ECDC) and mobility data sets.

To control COVID-19, many governments have declared mandatory or optional quarantines or are employing other policies. For simplicity, we used a 7-day window and transformed our daily mobility data into weekly data. We also performed missing value imputation using linear interpolation during this transformation. Our mobility data started on February 28, 2020 and ended on April 17, 2020, covering a total of 7 weeks in 26 countries (7 × 26 = 182 rows). For each country, using consecutive day pairs, we estimated the mobility averages of 9 variables (see Table 1).

**Table 1:** Mobility data obtained from Apple and Google.

Data		Google	Apple
Starting date		February 15, 2020	January 13, 2020
Ending date		April 11, 2020	April 21, 2020
Countries (n)		131	63
Subregions (n)		1710	89
**Variables**			
	1	Retail and recreation	Driving
	2	Grocery and pharmacy	Walking
	3	Parks	Transit
	4	Transit stations	N/A^a^
	5	Workplaces	N/A
	6	Residential	N/A

^a^Not applicable.

#### ECDC COVID-19 Data

In this study, our aim was to understand the relationships between reported mobilities and the dynamics of the COVID-19 outbreak. Several agencies, including the European Union, World Health Organization, and Johns Hopkins, offer up-to-date data aggregations of the number of cases as well as the number of deaths from over 150 countries. As one source of data, we used the ECDC data, which is updated daily on their website [26]. The data coverage was limited (no gender or age breakdowns, no data on the number of recovered patients or the number of tests conducted). We limited our analysis to the top 30 countries in terms of the number of cumulative cases. After the data transformations, we trimmed our data according to the starting and ending dates in Table 1.

#### Other Data Sets

During our study, to overcome the limitations of the ECDC COVID-19 data set (or similar data set providers), we also used several other data sets provided by individual countries such as the United States (the COVID tracking project by *The Atlantic* [27]), Belgium (the ECDC website [26]), and Turkey (the National Ministry of Health [28]). These data sets include the number of recovered patients on a daily basis.

### Methodology

To understand the relationships between limited mobility and the spread of COVID-19, we first established a target variable depicting the speed of the spread of the virus. The use of variables such as ”number of daily cases“ or ”number of daily fatalities“ was driven by many forces, such as ”natural course of the spread of the virus“ and ”limited mobility and other controllable effects.“ Because we were interested in measuring the actual changes in the diffusion of the spread, we decided to employ one of the most frequently used endemic models, the SIR model. Instead of looking at the case and fatality data, we investigated the relationship between the parameter changes of the SIR model and the changes in the mobility data set.

### The SIR Model

Pandemics are first characterized by a number referred to as the reproduction number, *R*_0_. This number approximately indicates the expected number of new infections caused by a single infection; hence, it has no unit. This is especially important during the early days of the spread of an infection. While *R*_0_<1 implies no epidemic, a greater *R*_0_ may indicate a pandemic of a larger scale. For instance, while seasonal influenza has an *R*_0_ of 1.3 [29], the *R*_0_ for COVID-19 is speculated to be around 2.2 [30,31]. During an outbreak, the trajectory of the number of infected people over time follows an approximately bell-shaped curve. Depending on the severity of the infection, health care systems are concerned with the peak of this curve to provide adequate health care services. The number *R*_0_ is simply obtained by multiplying the transmissibility per contact, the contacts per time unit, and the recovery rate.


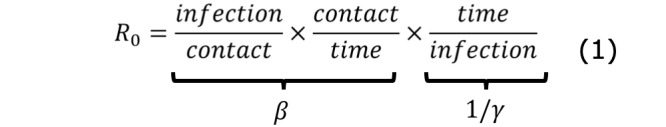


Perhaps the most frequently used model in epidemic models is the SIR model. The model categorizes individuals into three different compartments: susceptible (*S*), infected (*I*), and recovered (*R*). Therefore, it is called a compartmental model. Within the SIR model, the effective contact rate *β* controls the transition from compartment *S* to compartment *I*. This rate, which measures the number of new infections over time, may be influenced by interventions such as social distancing, wearing protective gear, or handwashing. The term *γ*, on the other hand, refers to the effective recovery rate. Therefore, a shorter average infectious period (1/*γ*) translates into a larger *γ* recovery rate. *γ* is strongly linked to the duration of the disease rather than to policy changes. Within the SIR compartment model, this value controls the move from compartment *I* to compartment *R*. The rates corresponding to intercompartment transitions can be written as a set of differential equations, as in equations 2-4 [32].

*dS/dt* = –*βSI/N* (2)

*dI/dt* = *βSI/N* – *γI* (3)

*dR/dt* = *γI* (4)

While this set of differential equations is self-explanatory, the parameter estimations, especially at the beginning of an outbreak, are usually not quite as straightforward. At the beginning of an outbreak, everyone may be considered as susceptible (*S ≈ N*), and *R*_0_ becomes *β/γ*. However, at later stages, *R*_0_ determines the size of the compartment *S* (*S* ≠ *N*); thus, it becomes numerically more challenging to calculate an estimate.

### Calculating γ

To determine a good approximation of the rate of recovery, we estimated the average number of days from case report to recovery. We used reported data available from three different countries: Turkey, Belgium, and the United States. By using a sliding window to investigate the correlation between the number of recovered cases and the number of new cases using a lag variable, we estimated the slide amount that maximizes the correlation between these two sets of numbers. While the results may depend on individual practices of the countries, our analysis consistently yielded a lag time of 7-8 days regardless of the country (see Multimedia Appendix 1 and Multimedia Appendix 3 for more details). Therefore, we chose to set *γ* at 1/7.5 = 0.133.

### Aggregating Reported Case Numbers for Analysis

ECDC reports the number of daily cases. Cases do represent infection; however, the number of infected cases on a given day does not simply equal the number of daily reported cases. While it may be more convenient to simply run the SIR model using daily case data, a more accurate approach involves estimating the number of infected individuals at a given time. Using our *γ* estimation of a 7.5-day average treatment window, we aggregated the daily case data to obtain an estimate of the number of active infections on each day.

### Fitting the SIR Model

Fitting a compartment model such as SIR is a numerical challenge. The curve fitting is usually achieved by solving a set of differential equations using the Runge-Kutta algorithm [33,34]. In our study, we were interested in how the effective contact rate of the infection, *β*, changes according to mobility. By fixing *γ* = 1/7.5, we sought to determine the value of *β* that minimizes the sum of squared errors.

Our mobility data started on February 28, 2020 and ended on April 17, 2020, covering a total of 7 weeks. For each country, using consecutive starting and ending weeks, we estimated the corresponding *β* of the SIR model (182 *β* values).

When estimating the *β* values, we used multilevel single linkage [35], Subplex (Nelder-Mead algorithm on the sequence of subspaces) [36], and Broyden–Fletcher–Goldfarb–Shanno quasi-Newton method [37] algorithms to check the consistency of the error-minimizing *β* parameter, and we reported the best value in terms of the mean squared error. All methods yielded identical *β* values, indicating the numerical stability of the fitted curve.

### Machine Learning Setup

As the last step of the extract, transform, load process, we merged the mobility data with the SIR model fits (*β* values) by adding a 1-week delay period to measure the effects of mobility on the overall fit of the model. Larger *β* values indicate a larger, faster spread (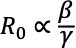). A graphical summary of the data merging and the study methodology is provided in Multimedia Appendix 2.

We investigated the relationship between *β* and the mobility factors by examining the predictive power of mobility with respect to *β*. Since the mobility factors were highly correlated, instead of training ordinary least squares regression models, which may raise multicollinearity concerns, we used the data to train a gradient boosted trees (GBT) model for regression.

GBT is a boosting ensemble machine learning approach that sequentially constructs a large number of decision trees; in each sequence, the algorithm reweights the training data based on the model performance in the previous sequence (giving a higher weight to instances with a more substantial error term). According to Hastie et al [38], GBT automatically disregards redundant features at any step due to its stepwise greedy strategy for selecting features in growing trees; hence, it is robust to multicollinearity.

Due to our limited sample size (N=130; 26 countries, 5 weeks per country), we employed a leave-one-out strategy to validate the GBT models. Each time, we used the algorithm to sequentially grow 2000 trees with a learning rate of 0.01 using 129 data points and tested the model on the remaining data point.

Moreover, to assess the importance of each single mobility variable in determining changes in *β*, we then examined the feature importance report provided by the GBT algorithm. For each predictor variable, the report provides a score indicating how valuable that variable was in the construction of the decision trees within the model. The more a feature is used to split the tree nodes, the higher its relative importance. A detailed discussion on how each score was calculated is provided in [38]. The results are described in the next section.

## Results

**Figure 1:**
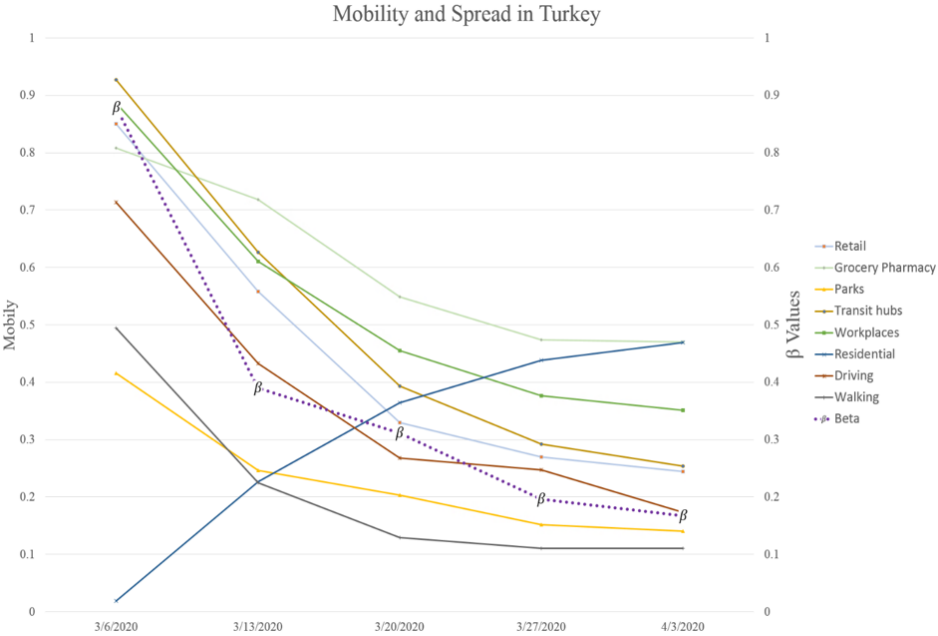
Mobility and spready in Turkey after lag is taken into account (the β values correspond to the week after the indicated date on the x-axis).

**Figure 2:**
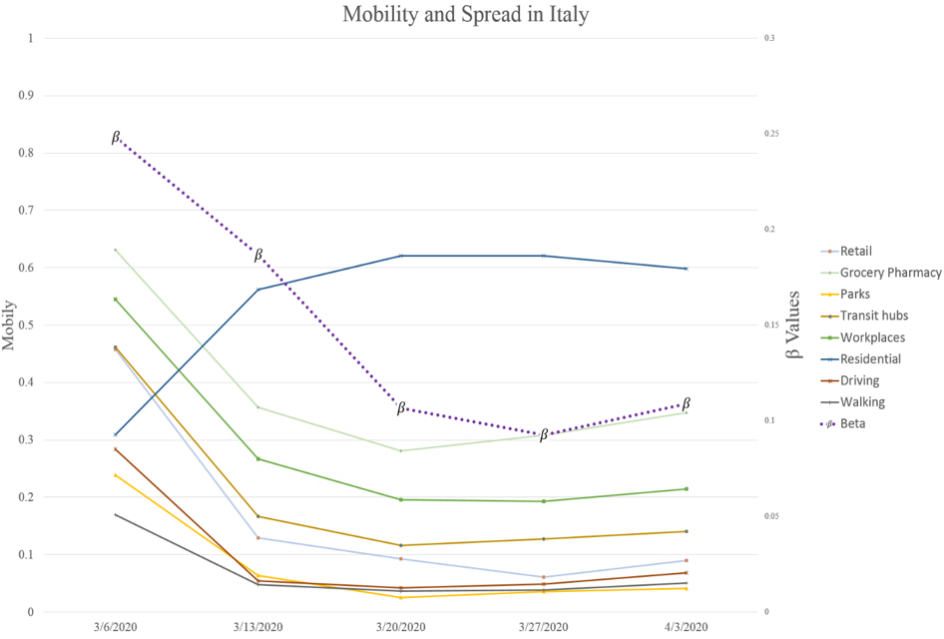
Mobility and spread in Italy after lag is taken into account (the β values correspond to the week after the indicated date on the x-axis).

While the mobility trends indicate lower mobilities, limiting mobilities resulted in increased residential mobilities across almost all countries. Figures 1 and 2 show a graphical depiction of our expected results. It can be observed that the *β* values mimic the mobilities of the earlier weeks. In the United Kingdom, for instance, while reduced mobility in earlier weeks resulted in a slower spread, a slight increase in mobility resulted in the growth of spread speed (larger *β*).

The GBT regression analysis results suggest that changes in mobility factors were able to explain around 47% of the variation in the COVID-19 transmission rate (*β*). The mean absolute error, mean squared error, and root mean squared error of the *β* predictions were 0.06, 0.005, and 0.072, respectively.

**Figure 3:**
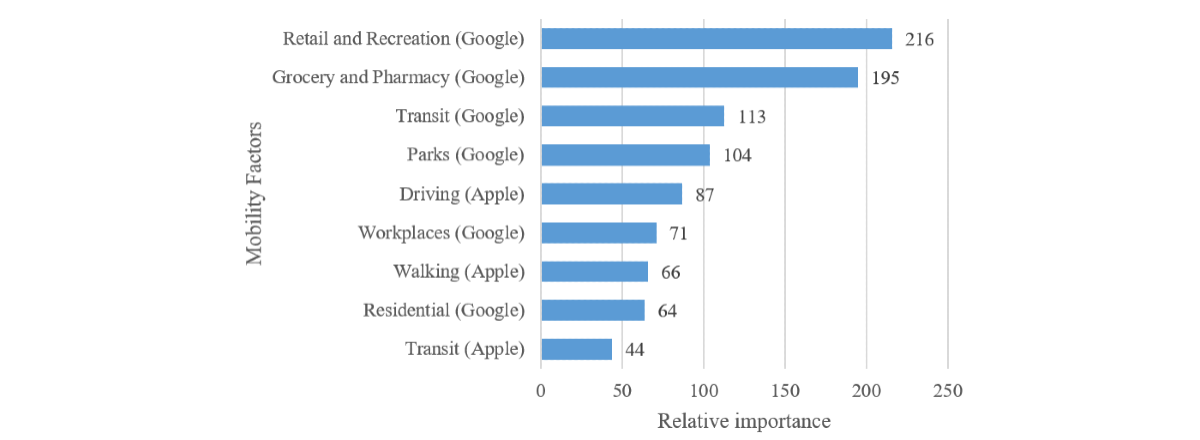
Relative importance of mobility factors in determining the COVID-19 transmission rate.

Figure 3 indicates the relative importance score of each mobility feature obtained from the GBT algorithm.

## Discussion

### Principal Findings

This study seeks to provide a more realistic and generalizable assessment of the effectiveness of social distancing interventions (reflected in mobility pattern changes) in controlling the spread of disease during a pandemic. Our results show that around 47% of the variation in the disease transmission rates is explainable by changes in mobility patterns resulting from enforcing of social distancing policies in the studied countries.

Also, as shown in Figure 3, changes of mobility in public places such as retail and recreation centers (eg, restaurants, cafes, theaters), grocery stores and pharmacies, transit hubs (eg, airports, bus stations, subways), and parks are the most important determinants of the disease transition rate. Additionally, interestingly, mobility in residential areas (the least public area) were found to be the second least relevant factor in predicting *β.* It should be noted that the transit mobility variable from the Apple data contained only zero values for 8/26 countries (31%). Because these values were not marked as missing in the original data set, we used them as provided. However, it is highly likely that these values were actually missing, in which case the Residential mobility variable would probably be the least important predictor of *β*. Overall, this justifies the government policies to enforce restrictions on travel, restaurants, and public events with the aim of controlling the spread of the disease.

Social distancing is an umbrella term that involves several different types of interventions, including case isolation, school closure, quarantine, distance working, and contact reduction in public places. Changes in mobility patterns, the effects of which were investigated in this research, can be considered as a surrogate measure of multiple social distancing interventions at the same time. The focus of other similar studies (mostly simulation-based) is on different combinations of these interventions, and different criteria were used to report the effects in those studies; therefore, comparing our results to theirs is challenging. For instance, Koo et al [14] used different combinations of *R*_0_ values and interventions and reported the mitigating effects in terms of the reduction in the number of infections (78%-99%), while Milne and Xie [15] examined several interventions sequentially and reported the mitigation role in terms of the reduction in the proportion of population infected (66%-24%). This study, meanwhile, uses the disease transmission rate *β* as the criterion to report the efficacy of social distancing.

From a theoretical viewpoint, this study contributes to the literature by proposing an approach for utilizing real data, as opposed to simulated numbers, to study the effects of various interventions at the time of an epidemic. We acknowledge that our results are highly affected by the lack of sufficient data (primarily due to the recency of the COVID-19 pandemic and the enforcement of social distancing policies); however, it still provides solid evidence of the effectiveness of social distancing. We argue that our results involve a considerably lower degree of uncertainty due to their reliance on real transactional data, which have already captured the complex dynamics of the epidemic. Also, since our data are not limited to a specific geographical area, our results should be more generalizable than those of similar studies, which are mostly limited to a certain area.

Different countries, due to differences in their public health policies and health care infrastructures, may be inconsistent in terms of the number of tests they perform and, consequently, in their reporting of the number of infections. However, we argue that since our approach only considers within-country changes for estimating the transmission rates, it is fairly robust to such inconsistencies. Also, we obtained identical *β* estimates from three different optimization algorithms, which shows that our estimates are robust with regard to the estimation methods as well.

Because we relied on real transactional data, we argue that this study provides a less noisy assessment of the efficacy of social distancing interventions than similar simulation-based studies. This is especially due to the complex nature of epidemics, which requires researchers who take a simulation approach to estimate several dependent parameters (eg, estimating the mortality rate depends on the number of infections, which itself depends on the transmission rate and the susceptible population), each of which are based on a set of assumptions that may be too simplistic in some cases; because each of those estimations may involve a reasonable error, this dependency leads to the introduction of a relatively high accumulated error in the whole study. Due to this complexity, most simulation-based studies only focus on the efficacy of a single social distancing policy (e.g., Earn et al [7] only examined school closure). Using real data, on the other hand, eliminates some sources of error by reducing the need for multiple estimations.

Moreover, due to the cross-national nature of the data, our results are more generalizable than those of similar studies that were mostly conducted in a single geographical area. Whereas countries may prefer to study the effects of their policies in their own situations, we argue that by fitting a single model to a multicountry data set, we mitigated the country-level idiosyncrasies in data; this provides policy makers with a clearer picture of how mobility is linked to the speed of disease spread.

From an empirical standpoint, in addition to providing supporting evidence for the effectiveness of social distancing policies, our study provides specific insights for policy makers as to which categories of locations and activities should be considered as top priorities for enforcing social distancing measures. Notably, our investigation revealed that mobility changes in highly public places such as restaurants, cafés, grocery stores, transit stations, and parks play more important roles in decreasing disease spread compared with workplaces or residential areas.

Additionally, our results suggest that reductions in driving mobility are relatively more important than changes in walking patterns in determining (decreasing) disease spread. This is also reasonable because the geographical span of driving mobility is normally far wider than that of walks; therefore, a susceptible person is subject to a higher risk of infection due to the potentially larger infected population residing in a wider area. This suggests that governmental restrictions on driving (especially long distances) can effectively reduce the number of new infections.

In addition to the relatively small sample size, another limitation of the present study is its reliance on highly aggregated data at the country level. Whereas this limitation is mainly due to the unavailability of granular mobility and COVID-19 data at the present time, we believe that replicating the proposed approach using a more granular mobility data set (in terms of the types of activities and categories of places) could reveal more interesting facts with regard to the effectiveness of specific social distancing policies. Therefore, we encourage future researchers to extend the present study as such data become available.

In the end, we believe that this study sheds light on the high potential of technology innovations in studying pandemics. Whereas we only took a retrospective approach by using historical geolocation data, a proactive approach that uses tracking technologies to identify people and locations at high risk could help governments and public health policy makers prepare for similar pandemics in the future. As a very recent effort, Google and Apple have announced a collaboration to implement a contact tracing system to send automatic mobile phone alerts to people who have recently been in close contact with people who tested positive for COVID-19 [39].

### Conclusion

Our analyses of real mobility and COVID-19 data provide substantial evidence of the significant mitigating role of social distancing interventions on disease transmission rates. Particularly, we have shown that controlling people's attendance and mobility in highly public places as well as enforcing driving restrictions are effective public health policies to help flatten the curve.

## Appendix

Multimedia Appendix 1 Merging mobility features and SIR model fits with a 7-day lag.

Multimedia Appendix 2 Graphical depiction of the data integration and analysis procedures.

Multimedia Appendix 3 Table S1. Determining the value of γ using a sliding lag window.

## Abbreviations

β: average transmission rate
COVID-19: coronavirus disease
R_0_: reproduction number
SIR: susceptible-infected-recovered
ECDC: European Centre for Disease Prevention and Control
