# Supplementary figures and images for: No Place Like Home: Cross-National Data Analysis of the Efficacy of Social Distancing During the COVID-19 Pandemic
Source: JMIR Public Health Surveill. 2020 May 28;6(2):e19862. doi: 10.2196/19862 (PMC7257477; doi:10.2196/19862)

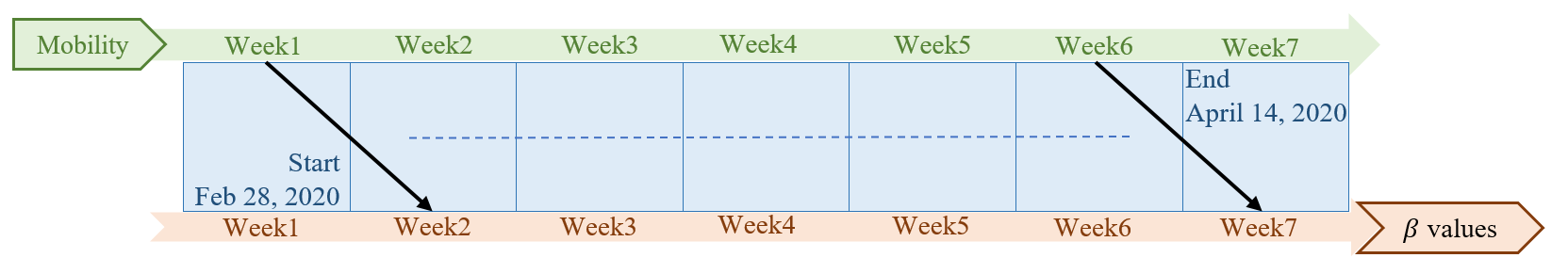

Supplement: Multimedia Appendix 1 [file publichealth_v6i2e19862_app1.png]

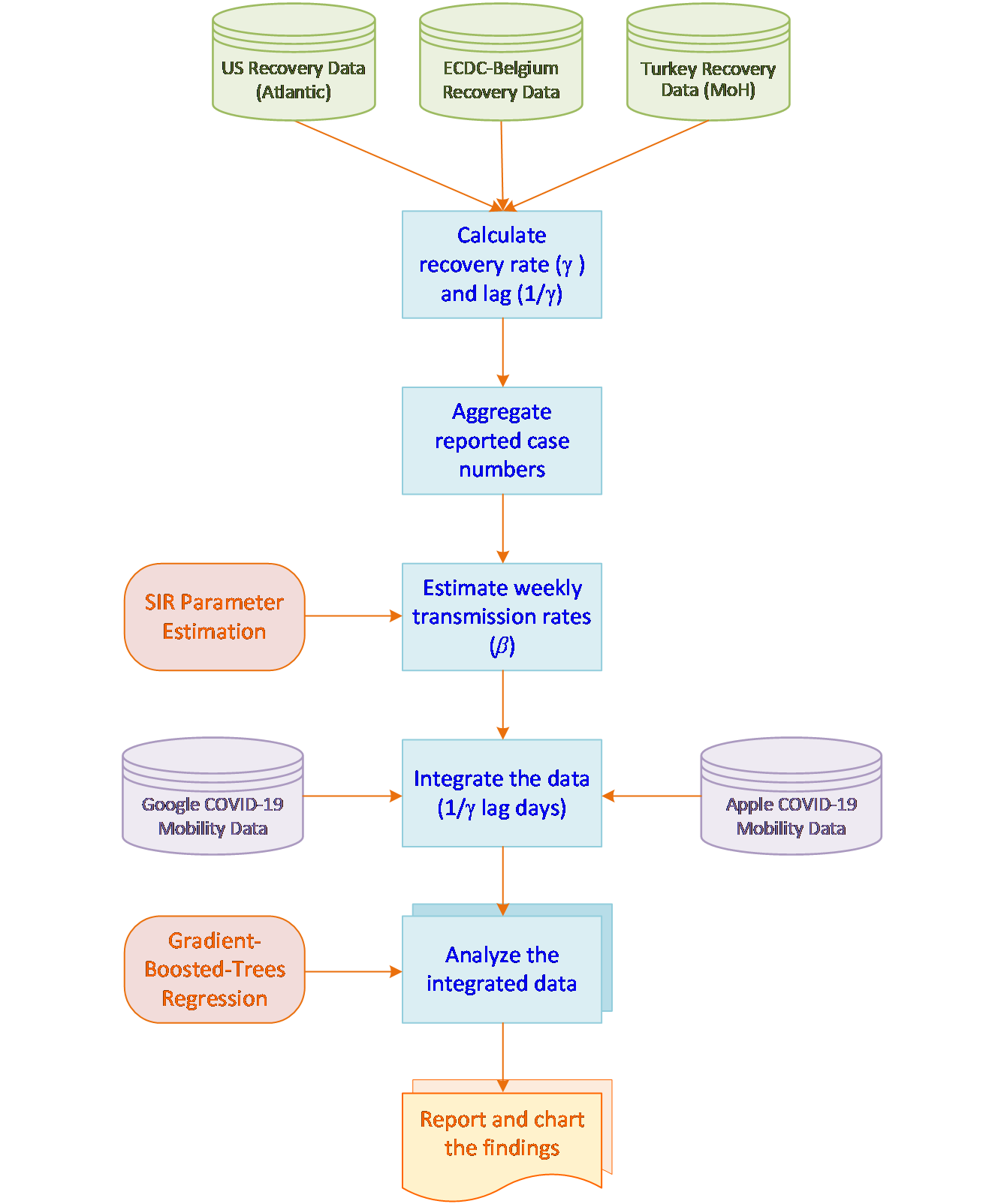

Supplement: Multimedia Appendix 2 [file publichealth_v6i2e19862_app2.png]
