# Supplementary material for: No Place Like Home: Cross-National Data Analysis of the Efficacy of Social Distancing During the COVID-19 Pandemic
Source: JMIR Public Health Surveill. 2020 May 28;6(2):e19862. doi: 10.2196/19862 (PMC7257477; doi:10.2196/19862)
Supplement: Multimedia Appendix 3 [file publichealth_v6i2e19862_app3.docx]

Table S1. Determining the value of $\gamma$ using a sliding lag window.

|  |  | Belgium | | |  |
| --- | --- | --- | --- | --- | --- |
| Lag (days) | Turkey | Brussels | Flanders | Wallonia | US |
| 1 | 0.658 | 0.462 | 0.515 | 0.049 | 0.9950 |
| 2 | 0.708 | 0.098 | (0.085) | (0.233) | 0.9947 |
| 3 | 0.731 | (0.016) | (0.149) | (0.487) | 0.9942 |
| 4 | 0.769 | (0.480) | (0.062) | (0.597) | 0.9939 |
| 5 | 0.810 | (0.466) | (0.510) | (0.658) | 0.9943 |
| 6 | 0.867 | 0.323 | (0.314) | (0.070) | 0.9953 |
| 7 | 0.897 | 0.182 | 0.461 | **0.852** | **0.9955** |
| 8 | **0.905** | **0.576** | **0.712** | 0.316 | 0.9951 |
| 9 | 0.902 | 0.226 | (0.026) | 0.136 | 0.9937 |
| 10 | 0.895 | (0.080) | (0.302) | (0.026) | 0.9917 |
| 11 | 0.900 | (0.422) | (0.252) | (0.465) | 0.9896 |
| 12 | 0.879 | (0.333) | (0.311) | (0.697) | 0.9874 |
